# Optimization of the Process of Eliminating Microorganisms Harmful to Human Health and Threatening Objects Isolated from Historical Materials from the Auschwitz-Birkenau State Museum in Poland (A-BSM) Collection with the Use of Ethanol in the Form of Mist

**DOI:** 10.3390/ma16072700

**Published:** 2023-03-28

**Authors:** Anna Wawrzyk, Marzena Dymel, Krystyna Guzińska, Piotr Cywiński, Aleksandra Papis, Adam Konka, Iga Wawrzyk-Bochenek, Sławomir Wilczyński

**Affiliations:** 1 Auschwitz-Birkenau State Museum, Więźniów Oświęcimia 20, 32-603 Oświęcim, Poland; 2 Silesian Park of Medical Technology Kardio-Med Silesia in Zabrze, M. Curie Skłodowskiej 10C Str., 41-800 Zabrze, Poland; 3 Lukasiewicz Research Network-Lodz Institute of Technology, M. Sklodowskiej-Curie 19/27, 90-570 Lodz, Poland; 4 Department of Basic Biomedical Science, Faculty of Pharmaceutical Sciences in Sosnowiec, Medical University of Silesia, Kasztanowa 3, 41-205 Sosnowiec, Poland

**Keywords:** ethanol mist, historical object, microorganisms, disinfection

## Abstract

The aim of the study was to assess the biocidal effectiveness and the effect of 80% and 90% ethanol applied in the form of mist on the surface of textile materials from historical A-BSM objects. The microorganisms used for the tests, namely, *Cladosporium cladosporioides*, *Aspergillus niger* and *Penicillium chrysogenum*, were isolated from the surface of textile objects in the A-BSM. *Bacillus subtilis*, *Staphylococcus aureus*, *Aspergillus flavus* and *Aspergillus niger* were also used from the American Type Culture Collection (ATCC). Fabric samples were inoculated with microorganisms at a concentration of 10^5^–10^6^ CFU/ml. Ethanol in the form of mist was applied in concentrations of 80% and 90%. Airbrushes VL 0819 and VE 0707 were used for this purpose, where the pressure was 0.2 MPa and the PA HEAD VLH-5 nozzle with a tip of 1.05 mm in diameter was used. In order to achieve more effective disinfection after applying the ethanol mist, samples were stored in PE foil in the conditions of 21 °C ± 1 °C for 22 ± 1 h. After applying the ethanol mist, changes in the properties of the materials were assessed using scanning electron microscopy (SEM). The reduction in the number of microorganisms on modern cotton fabric after the use of ethanol in the form of mist at concentrations of 80% and 90% ranged from 93.27% to 99.91% for fungi and from 94.96% to 100% for bacteria, except for 74.24% for *B. subtillis*. On the historical fabric, after the time of application of 90% ethanol was shortened to 4 s, the microorganisms were reduced by over 99.93% and *S. aureus* was completely eliminated. After applying the tested disinfection technique, no changes in fiber morphology were observed on the surface of the model and historical cotton.

## 1. Introduction

Konzentrationslager Auschwitz-Birkenau was established in 1940 by the German Nazi regime. The camp served as a place of slave labor and starvation, quickly becoming the largest center of immediate and mass extermination of the population.

In order to preserve the memory of the victims, the Auschwitz-Birkenau State Museum (A-BSM) was founded at the side of the former extermination and concentration camp. The activity of this museum focuses on securing the remains of the former camp, including personal belongings found after liberation, often constituting the only evidence of the presence of people in this place. A large group of objects, the so-called looted property, comprise items containing fabric in their composition. These include clothing, shoes, suitcases and orthopedic supplies. Most of the objects are in a bad condition. This is the result of the methods of serial mass production, poor quality materials and their intensive usage. However, the main factor influencing the degradation was the way these objects were used and treated in the camp. Taken from their owners, they were stored in piles in outdoor conditions. Frequently searched, and therefore, especially destroyed, they awaited a decision on their next use: transport to the Reich, processing or deliberate destruction.

One of the most numerous groups of objects in the Museum Collections is shoes. Footwear is usually made of several different materials. Apart from leather, textile materials were most often used. The fabric recognized in the shoes is primarily cotton canvas of various thicknesses, as well as wool felt. The laces are also textiles. Suitcases are important objects that enable obtaining information about the owner. Among the surviving, cardboard with a paper finish predominate, to rarer but also forming a large group, those with textile components. The fabric in the suitcases was used both as a lining and as a material for the inner straps holding the contents of the suitcase interior. The fabric was also used as an external cladding, but often more as a carrier of synthetic coatings imitating other materials. The most commonly recognized fabrics are those made of cotton and linen canvas. Orthopedic supplies were also selected for the tests described in this article. During the selection of Jews carried out on the ramp, the SS doctors sent people with physical disabilities, among others, to immediate death in the gas chambers. The property left by them, including all various types of prostheses, has a fabric that was supposed to facilitate the regulation of body temperature.

Through its activities, the museum takes care of the proper storage of the collections and archives remaining from the camp. All objects and their elements require protection against the natural processes of biodeterioration. Biodeterioration is any undesirable change in the material caused by the activity of microorganisms [1].

Microorganisms originating from the ground that are carried by the air movement on historical objects or brought by visitors gradually colonize their surfaces and, through enzymatic activity, cause decomposition to simple compounds. Microorganisms on the surfaces of museum objects produce cellulases, amylases and proteases, which then start biodeterioration. As a result of microbial decomposition, stains, deposits, structural weakness and color changes may appear on historical objects [2]. Microbes in museum conditions often tolerate unfavorable environmental conditions. They are resistant to low and high pHs, changes in temperature and humidity, and a small amount of organic substances is enough for them to survive and develop. Particularly susceptible to decomposition are organic materials made of wood, leather, paper and fibers of plant and animal origin, such as cotton, wool or silk. The mechanism of microbial degradation of organic materials is based on the use of lignocellulolytic enzymes in the case of wood and paper, cellulases, keratinases and esterases in the case of textiles [3,4].

One of the stages of securing historical objects is their decontamination. Currently, various methods of disinfection are used in museums. These include washing with alcohol, spraying with quaternary ammonium compounds or fogging with ethylene oxide. Many of the biocidal substances used have an adverse effect on the disinfected object. For example, they can change the color and pH, and thus, the structure [5]. The decontamination effect may be counterproductive and cause accelerated aging, which is not acceptable for historical materials.

An important aspect of the process of disinfection of historical objects is the health and safety of people performing conservation work. Microorganisms are harmful, but most of the biocidal substances used so far have also been a serious threat to human health. One such compound is ethylene oxide (ETO), which is very effective and safe for most museum objects, but is toxic and carcinogenic to humans [6].

When taking care of the proper protection of the enormous value of historical objects, the Auschwitz-Birkenau Museum strives to find the best means of disinfection that are safe for people and objects. Therefore, an important element of the activity is the search for new methods of decontamination that can be used on historical materials. Works at the A-BSM are based on new disinfection methods, previously not used in museology but previously implemented in medicine, e.g., the use of a diode laser, which is used to disinfect root canals and implants, and VHP (vaporized hydrogen peroxide), which is used to decontaminate surfaces in hospitals. However, the use of new techniques in museology requires the optimization of doses due to the different structures of the degraded surface.

In this study, an attempt was made to use ethanol to disinfect historical materials. Ethanol is a widely known disinfectant in medicine and is most often used in liquid form. Its action is based on the denaturation of microbial proteins. A liquid ethanol solution can negatively affect the disinfected historical object by dissolving compounds that are sensitive to contact with aqueous solutions. Tests performed on historical paper confirmed that ethanol from 60% to 90% destroys cells of bacteria, fungi and viruses. One molecule of ethanol contains an -OH group, which makes it a substance with a high oxidation–reduction potential, and thus, it can react with virtually any chemical substance, which is undesirable in historical objects [7]. Due to the fact that ethanol in liquid form soaks surfaces, making objects vulnerable to damage, deformation or discoloration, but at the same time it is safe for use by conservators, ethanol was tested using a much more delicate form of application. It was assumed that a small amount of water would not remain on the surface for a long time, which would not increase the humidity of the object, and that ethanol would have a biocidal effect and would also evaporate.

**Figure 1:**
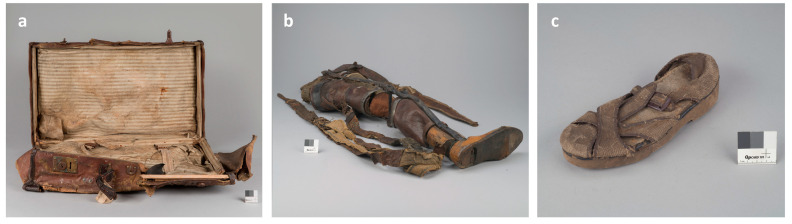
Textile objects from Auschwitz-Birkenau State Museum: (**a**) suitcase; (**b**) prosthesis; (**c**) shoe (sources: (**a**) Adam de Sas Topolnicki, (**b**) Sebastian Mrozek, (**c**) Jerzy Bestyński).

Some of the objects from the PMA-B collections, whose components are made of cotton fabrics and leather, require preservation (Figure 1). The travel suitcase (Figure 1a) is made of cardboard covered with a decorative coating based on cellulose nitrate, and the inside is lined with linen fabric in parallel stripes. The right leg shin prosthesis (Figure 1b) is made of wood and leather, and the carrying belt is made of fabric. The prosthesis shows signs of many repairs made by the owner, and the fabric is mechanically very weak. The sandal (Figure 1c) has a wooden sole and a cardboard insert, is fastened with a metal buckle and is mostly made of a coarse weave fabric.

In 2018, the results of research conducted to identify microorganisms found on cotton fabrics in the A-BSM were published. At that time, 13 species of bacteria were identified: *Bacillus atrophaeus*, *Bacillus cereus*, *Bacillus licheniformis*, *Bacillus megaterium*, *Bacillus simplex*, *Bacillus subtilis*, Enterobacter sp., *Lysinibacillus fusiformis*, *Micrococcus luteus*, *Paenibacillus provencensis*, *Pseudomonas stutzeri*, *Psychrobacillus psychrodurans* and *Staphylococcus cohnii*. In addition, 12 species of fungi were identified: *Alternaria alternata*, *Aspergillus flavus*, *Aspergillus niger*, *Chaetomium globosum*, *Cladosporium cladosporioides*, *Epicoccum nigrum*, *Fusarium poae*, *Mycelium sterillium*, *Penicillium chrysogenum*, *Rhizopus nigricans*, *Stephanoascus cifferrii* and *Trichoderma viride* [6]. In other studies, Gram-positive bacilli, Gram-positive cocci, Gram-negative bacteria, filamentous fungi, yeast-like fungi and Candida yeasts were isolated from cellulosic materials collected in the A-BSM. *B. subtilis*, *S. epidermidis*, *A. flavus and A. niger* were detected in all historical textiles examined [8].

After analyzing the obtained results, the following strains were selected for further work aimed at the use of an effective disinfectant for museum facilities: *Bacillus subtilis*, *Staphylococcus aureus*, *Aspergillus niger*, *Aspergillus flavus*, *Alternaria alternata*, *Cladosporium cladosporioides* and *Penicillium chrysogenum*.

The species were selected for their harmful effects on human health, destructive effects on objects and the highest frequency of occurrence. Fungi of the genera Aspergillus, Alternaria, Cladosporium and Penicillium can cause low-intensity chronic inflammation in humans resulting from continuous exposure to mold fungi in the work environment; they can also contribute to the development or exacerbation of respiratory diseases and affect the severe courses of diseases caused by respiratory infections [9].

*Staphylococcus aureus* can pose a threat to human health, with it being transmitted by air and dust, or through direct contact with exhibits on which it lives, causing allergies and purulent infections of the skin [10].

Microorganisms with the ability to decompose cellulose and produce acids that contribute to the progress of biodeterioration on objects (*B. subtilis*, *A. alternata*, *A. flavus*, *A. niger*, *C. cladosporioides* and *P. chrysogenum*) were also selected for the study [11].

*Bacillus subtilis* is not a typical human pathogen but it was isolated several times from immunocompromised people with bacteremia, endocarditis, pneumonia and sepsis. At the same time, it is a spore-forming microorganism that is very difficult to eliminate from the environment and very resistant to external factors [12].

Depending on the material from which the historical object is made, the microorganisms on its surface can react differently to disinfectants. Due to the good effectiveness of ethanol in liquid form, as demonstrated on medical surfaces and other abiotic surfaces, in this study, the effect of ethanol was tested using a gentle method of application, i.e., in the form of mist.

All conservation works, including decontamination, must ensure that they do not adversely affect the surface structure of the historical object. For this purpose, an analysis of the surface before and after the application of a new disinfection technique should always be performed. In this work, SEM imaging was used, which was previously applied in the A-BSM to study the structure of cellulose fabrics and objects [8,13].

## 2. Materials and Methods

### 2.1. Research Objects

The microorganisms used for testing were isolated from shoes, suitcases and prostheses in the A-BSM collection, which included fabric items. The microorganisms were obtained in previous studies, deposited in cryobanks of the A-BSM laboratory and, after multiplication, were used in this work.

Tests on the effectiveness of the disinfecting effect of ethanol (Chempur, Piekary Śląskie, Poland) were carried out on cotton (SDC Enterprises Limited, Pickwick Mill, UK) with a weight of 100 g/m^2^, hereinafter referred to as model cotton. The model fabric was cut to dimensions of 10 × 10 cm. Samples were sterilized before the decontamination effectiveness test (121 °C, 20 min).

Tests of the effectiveness of ethanol were also carried out on historical cotton taken from the inside of the lid of a suitcase from the first half of the 20th century that was located in the A-BSM collection.

### 2.2. Biocidal Efficacy Tests

#### 2.2.1. Microbial Strains

Strains from the American Type Culture Collection (ATCC) were used to test the effectiveness of ethanol, i.e., *Staphylococcus aureus* ATCC 6538, *Bacillus subtilis* ATCC 6635, *Aspergillus niger* ATCC 6275, *Aspergillus flavus* ATCC 9643 and strains isolated from the museum objects: *Cladosporium cladosporioides*, *Alternaria alternata* and *Penicillium chrysogenum*.

Fungi were inoculated on Sabouraund’s agar medium (SDA) with 4% glucose (BTL, Łódź, Poland). All plates were incubated at 25 ± 2 °C for 5 days. The cultures were suspended in sterile water so as to obtain a density of about 10^5^–10^6^ CFU/sample on the fabric. Then, it was shaken with glass beads, filtered through sterile cotton and centrifuged three times. The obtained suspension was diluted each time and the density was determined using the plate method.

Bacteria were inoculated on standard count agar (PCA) (BTL, Łódź, Poland). All plates were incubated at 30 ± 2 °C for 2 days. The cultures were suspended in sterile water and assayed on the McFarland scale. Then, the suspensions were diluted in peptone water and determined using the plate method so as to obtain a density of about 10^5^–10^6^ CFU/sample on the fabric.

The suspensions prepared in this way were applied to fabrics and then dried under sterile conditions.

#### 2.2.2. Application of Ethyl Alcohol Mist

The characteristics of the examined samples of historical materials after treatment with ethanol in the form of mist were subjected to standard assessments of the state of preservation performed by conservators in the PMA-B laboratories. Such an assessment was made at the stage of preliminary tests. The samples received a positive evaluation, i.e., no changes in color and structure were observed.

After drying, ethanol (Chempur, Piekary Śląskie, Poland) was applied to the inoculated material using two-action airbrushes VL 0819 and VE 0707 from Paasche (Kenosha, WI, USA) at a pressure of 0.2 MPa and using a PA HEAD VLH-5 nozzle with a tip of 1.05 mm diameter.

The biocidal effectiveness of short- and long-acting ethanol in the form of mist was assessed. When assessing the short-term effect of ethanol, the weight of the applied solution depended on the concentration of alcohol and was 0.2–1.0 g per 100 cm^2^. The application time was set at 4–16 s/100 cm^2^. The cotton was then dried. In order to test the long-term effect of ethanol, after activities analogous to the assessment of the short-term effect of ethanol, the time of contact of microorganisms with ethanol was additionally extended, i.e., after application, the cotton was wrapped in PE foil and dried after 22 h.

#### 2.2.3. Washing and Labeling of Microorganisms

The fabrics were dried and the microbes were washed out of the fabric by shaking for 5 min at 250 rpm in 100 mL 2% Tween peptone water. Decimal dilutions were made and sown on the substrate. Fungi were incubated on SDA medium for 3 days at 25 °C and bacteria were incubated on PCA medium for 24 h at 37 °C.

#### 2.2.4. Counting the Number of Microbes

The number of microorganisms was calculated using the following formula:

$$M=\frac{\sum C}{v\left(n_{1}+0.1n_{2}\right)d}\times 100$$

where

*M*—number of bacteria/fungi on the sample (CFU/sample);

*C*—sum of colonies on all plates from the counted dilution;

*V*—volume of inoculation applied to each plate in ml;

*n*_1_—number of plates from the counted dilution;

*n*_2_—number of platelets from the second counted dilution;

*d*—the dilution factor corresponding to the calculated dilution.

In case no colonies were detected on two plates, the result was reported as <100 or logarithmic scale < 2. This means that no microbes were present or were below the limit of detection.

The percentage and logarithmic reductions were calculated from the obtained number of microorganisms.

The reduction percentage was calculated using the following formula:

$$\mathrm{Reduction}\;\%=\frac{W-T}{W}\times 100$$

The logarithmic reduction was calculated using the following formula:

$$\mathrm{Reduction}\;\log_{10}=\log\;T-\log\;W$$

where

W—number of bacteria on the model cotton without alcohol applied at the same time as samples with ethanol applied;

T—the number of bacteria on a model or historical sample after treatment with ethanol.

### 2.3. Surface Morphology Analysis

Scanning electron microscopy (SEM) was used to check whether ethanol caused unfavorable changes in the surface morphology of the disinfected model and historical fabric. For this purpose, the samples were mounted on aluminum holders using self-adhesive carbon discs. To obtain better topographical contrast and to eliminate static electricity during scanning, the samples were covered with a layer of gold using EM SCD005 sputtering (Leica, Wetzlar, Germany). Imaging at magnifications of 100×, 500× and 5000× was performed with an FEI Quanta 3D FEG scanning microscope (Thermo Scientific, Waltham, MA, USA). SEM images were taken in a high vacuum with a low accelerating voltage.

The photos presented in this paper were taken of samples after the application of 90% ethanol immediately after application and after 22 h of exposure to vapors.

## 3. Results

### 3.1. Ethanol Application Optimization

In the first stage of the research, the method of applying ethanol to the surface of model cotton was established. The airbrushes VL 0819 and VE 0707 from Paasche were selected, which allowed for the most even spray of ethanol in the form of mist, which was visually assessed on the basis of visible traces of fabric soaking.

Then, the distance of the airbrush from the fabric was selected so as to obtain a possibly even application with low wetting and the smallest possible losses resulting from the dispersion of ethanol. The best efficiency was obtained with a distance of 16 cm from the airbrush to the fabric. The size of the fabric on which the ethanol was applied was also standardized, which ultimately amounted to 10 × 10 cm. An additional parameter that was introduced in order to obtain repeatability of the method was the time of application to the fabric.

The tests were carried out for 9, 10 and 15 s, depending on the concentration of ethanol and the stage of the study; in order to reduce costs, in the last stage of the research, the application time of 4 s was also tested, which also turned out to be effective for most microorganisms. At the stage of optimizing the parameters of ethanol mist application, the tests were performed on a model cotton fabric, which was not a museum object, in three repetitions in order to check the repeatability of the process.

### 3.2. Efficacy of Short-Acting Ethanol

Two concentrations of ethyl alcohol in the form of mist were tested against five strains of microorganisms. Table 1 presents the results for two microorganisms isolated from the museum objects, namely, *Cladosporium cladosporioides* and *Alternaria alternata*, and three microorganisms from the ATCC collection, namely, *Staphylococcus aureus*, *Bacillus subtillis* and *Aspergillus niger*. Ethanol mist was applied to the samples under constant conditions: 16 cm airbrush-to-sample distance and spray times of 9 s (90%) and 10 s (80%) and 15 s for *A. alternata* at both concentrations. In the case of the *A. niger* fungus, the mist application time was extended to 15 s to achieve a comparable mass of ethanol deposited due to the use of the VE 0707 airbrush.

For each of the tested microorganisms, a relationship was observed between the amount of alcohol applied and its concentration. In all tested cases, this amount was higher for 80% ethanol (0.64 ± 0.14 g) than 90% (0.47 ± 0.11 g), which was related to the rate of evaporation. In addition, the amount of alcohol applied to *A. alternata*, despite maintaining constant application conditions, was slightly higher than for the other strains.

The reduction in most microorganisms after the use of ethanol mist ranged from 93.62% to 99.91%. Only *B. subtillis* showed a much lower reduction, i.e., 14.9%. For ethanol at a concentration of 80%, the lowest reduction percentage of 93.63% was shown for *A. niger*, and the highest reduction was achieved for *A. alternata* at 99.21%. In the case of 90% ethanol mist, *C. cladosporioides* had the highest reduction at 99.91%, and the worst was *A. niger*, i.e., by 93.27%.

For the environmental strains, a reduction in *C. cladosporioides* of 3.06 log was obtained for the use of 90% ethanol and 2.07 log for 80%. On the other hand, *A. alternata* was reduced by 2.06 logarithms for an alcohol concentration of 80%.

Microorganisms were not completely eliminated from the material in any case.

The biocidal effectiveness of ethanol mist against the Gram-(+) motile Bacillus subtilis was evaluated using a wider range of ethanol concentrations: 60%, 70%, 80% and 90%. The results are presented in Table 2.

*B. subtillis* was reduced the most (41.49%) after the application of the 60% solution, and the least (14.90%) after the application of 80% ethanol. In each case, a satisfactory reduction was not achieved.

**Figure 2:**
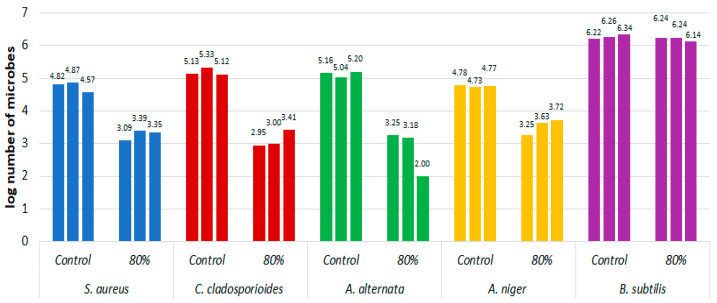
Effect of short-term exposure to 80% ethanol in the form of mist on the log decrease in the number of microorganisms on the model fabric compared with the control not treated with the disinfectant.

Figure 2 shows the log_10_ of the number of microorganisms on the model fabric treated with 80% ethanol in a coating of 0.64 ± 0.14 g and on the fabric not treated with alcohol for the following microorganisms: *C.cladosporioides*, *A.alternata*, *B.subtilis*, *S.aureus* and *A. niger*. The test was performed in three repetitions. All strains showed a decrease in microorganisms under the influence of alcohol, except for *B. subtilis*, where the number of cells per sample did not decrease for all replicates. The greatest difference between the control sample without application and the sample after the application of ethanol was shown for the fabric inoculated with *C. cladosporioides*.

**Figure 3:**
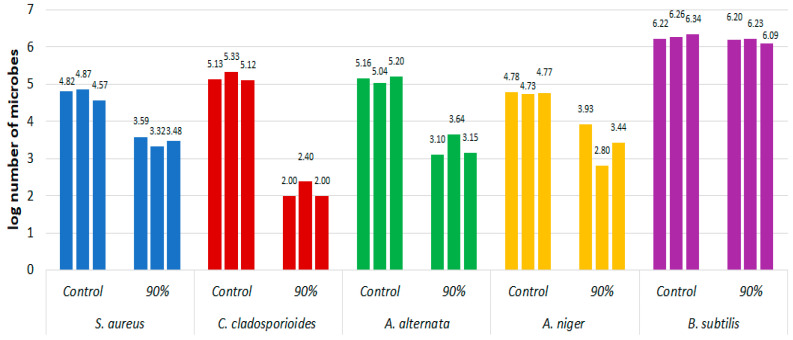
Effect of short-term exposure to 90% ethanol in the form of mist on the log decrease in the number of microorganisms on the model fabric compared with the control not treated with the disinfectant.

Figure 3 analogously shows the log_10_ of the number of microorganisms on the model fabric treated with 90% ethanol with an application of 0.47 ± 0.11 g and on the fabric not treated with alcohol for the same microorganisms.

### 3.3. The Effectiveness of Long-Acting Ethanol

In order to further optimize and achieve an increasing reduction in the number of microorganisms, the duration of exposure to ethanol was extended. A similar method but using ethanol vapor in museums to disinfect paper was used [14].

Fabrics contaminated with microorganisms were disinfected with ethanol in the form of mist using an airbrush, then tightly wrapped in PE foil and stored at 21 °C ± 1 °C for 22 ± 1 h.

The first tests were carried out for the strains *S. aureus*, *B. subtilis*, *A. flavus* and *A. niger* with the same conditions of ethanol application, i.e., a distance of 16 cm and application time of 10 s for 80% solution and 9 s for 90%. The results are shown in Table 3.

For the strains *S. aureus*, *A. flavus* and *A. niger*, the degrees of logarithmic reduction were above 2 log, and the percentage reductions were above 99.9%. *B. subtillis* was reduced at the level of 73–74%.

Due to the protection of museum objects against the adverse effects of ethanol and the economy of the decontamination process itself, an attempt was made to reduce the amount of alcohol applied. The application time was reduced to 4 s, which allowed for applying 0.36 ± 0.07 g of the 80% solution and 0.28 ± 0.04 g of the 90% solution. Table 4 shows the results. The reductions obtained for the strains *of S. aureus*, *A. flavus* and *A. niger* were the highest and ranged between 99.9% and 100%.

In a further part of the study, tests were carried out using historical cotton fabric from the A-BSM collection. The tests were performed with 90% ethanol acting on microorganisms for 22 h in one repetition. The alcohol was deposited for 4 s, which resulted in a deposit of 0.27 g to 0.30 g. *S. aureus* ATCC, *A. alternata* ATCC, *A. flavus* ATCC and *A. niger* ATCC, as well as the environmental strains of *A. niger* and *P. chrysogenum*, were used. The obtained results are presented in Table 5.

On the historical material, the reduction in microorganisms was between 99.87% and 100%. Similar results were obtained on the model material. Among the fungi, *Penicillum chrysogenum* isolated from museum objects was reduced the least, and *Aspergillus flavus* was reduced the most. *S. aureus* was eradicated.

For all tested microorganisms, a total logarithmic reduction was obtained, which, depending on the growth in the control sample, ranged from 2.88 to 4.41.

### 3.4. Analysis of Changes on the Surface of the Disinfected Model and Historical Material

**Figure 4:**
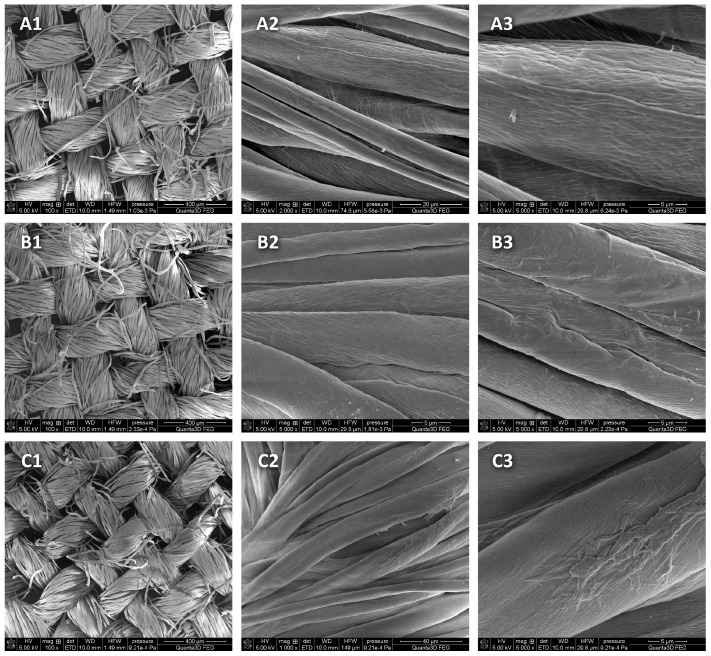
SEM micrograph showing the topographies and surface morphologies of the samples: (**A1**–**A3**) model cotton—control; (**B1**–**B3**) model cotton—after the application of 90% ethanol in the form of mist and direct drying; (**C1**–**C3**) model cotton—after the application of 90% ethanol in the form of mist and drying after 22 h of storage in ethanol vapor. Enlargements: (1) 100×, (2) 500× and (3) 5000×.

SEM imaging was performed to assess the changes in the topography and surface morphology under the influence of 90% ethanol in the form of mist on the model fabric. Model cotton was selected, on which 90% ethanol was applied and dried immediately after application or after 22 h of storage in a closed package (Figure 4).

SEM examinations of model cotton samples after the use of 90% ethanol in the form of mist showed no differences (losses) in the structure of the tested material in the cotton samples subjected to disinfection processes compared with the control sample.

**Figure 5:**
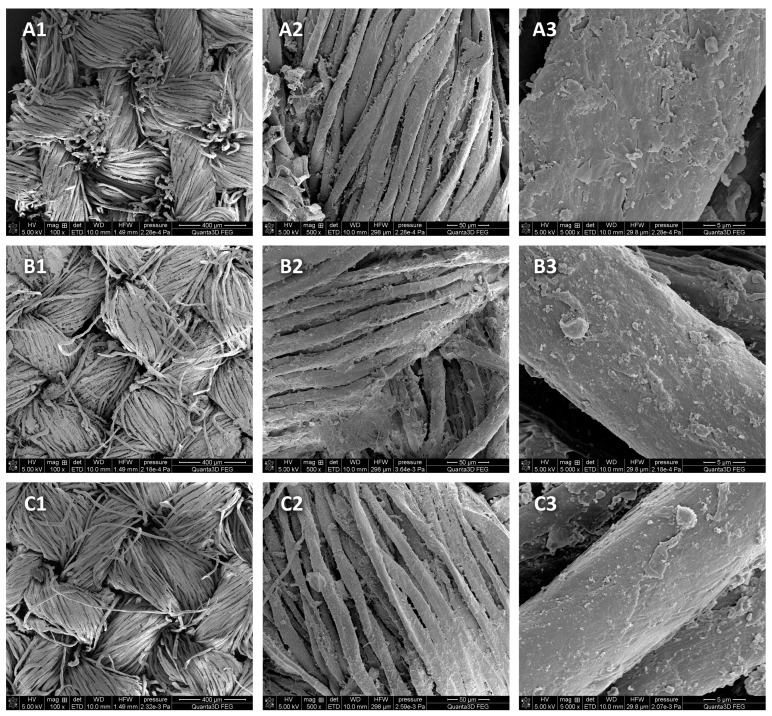
SEM micrographs showing the topographies and surface morphologies of the samples: (**A1**–**A3**) historical cotton—control; (**B1**–**B3**) historical cotton—after the application of 90% ethanol in the form of mist and direct drying; (**C1**–**C3**) historical cotton—after the application of 90% ethanol in the form of mist and drying after 22 h of storage in ethanol vapor. Enlargements: (1) 100×, (2) 500× and (3) 5000×.

Changes were also not shown on the historical fabric after the use of 90% ethanol in the form of mist dried immediately after application and after 22 h of storage in a closed package (Figure 5).

## 4. Discussion

In 2018 and 2020, data on microbiological contamination on cotton fabrics in the A-BSM were published. Among the microorganisms, microorganisms that may adversely affect the condition of objects and human health were found [8].

The presence of certain bacteria and fungi on the surfaces of historical objects may pose a threat to the health of people working in museums [15,16].

In order to prevent the spread of microorganisms, various methods of surface decontamination are used in the A-BSM. Disinfection with quaternary ammonium salts, liquid alcohol solution, VHP (vaporized hydrogen peroxide), EtO (ethylene oxide) and diode laser irradiation were implemented so far. Each of these methods is dedicated to other facilities that differ in the size of the area to be decontaminated. VHP and EtO are used to decontaminate objects as a whole, while the laser is used to eliminate microorganisms from very small spots, the so-called biodeterioration in the initial phase. The ethylene oxide used so far turned out to be carcinogenic, and thus, it was eliminated from use in the A-BSM. The A-BSM is constantly looking for techniques that will enable disinfection limited to the place with visible traces of biodeterioration.

The area from which the A-BSM disinfection techniques were adapted for museology is medicine, where they are already well-known and researched [17]. The medical decontamination technique with the use of VHP implemented in the A-BSM, after adjusting the doses of VHP to concentrations safe for historical objects, allowed for achieving a reduction in microorganisms on historical cardboard at the level of over 99% [13].

The A-BSM successfully performs disinfection with a diode laser, for which in the medical area, a reduction of bacteria and fungi from 60% to 100% was achieved on various materials [18,19,20,21,22]; on historical collagen material in the A-BSM, a reduction of 78–92% was achieved, and on cellulose, 90–100% was achieved [8,23,24].

In this study, an attempt was made to check whether ethanol, which has been successfully used in medicine so far, can be used to disinfect museum objects that are highly sensitive to biocidal substances [25].

In tests with 85% liquid ethanol, a comprehensive bactericidal effect was demonstrated in 15 s [26]. The best effect was found at concentrations of 80–90% ethanol. Sauerbrei published data showing that the optimal germicidal concentration of ethanol, which inactivates spores, is 80–85% with an exposure time of 30 s. A concentration of 80–90% ethanol has a virucidal effect, which also includes enveloped viruses and adeno-, noro- and rotaviruses. It also showed that the concentration of 100% ethanol does not have a safe bactericidal and virucidal effect [27].

Koshiro and Oie demonstrated complete inactivation of Gram-negative and Gram-positive bacteria, except for *Staphylococcus aureus*, after the application of 99.5% ethanol [28].

The use of ethanol in liquid form was also tested on artifacts. A 70% EtOH solution had a mild positive effect on chemical stabilization and did not cause discoloration of the historical paper [25].

Chloe et al. demonstrated the effectiveness of fungal growth inactivation on historical gelatin–silver prints depending on the temperature and contact time of ethanol with the fungus. The four tested strains were inactivated after only two hours of exposure to water and ethanol vapors. They also showed that direct contact tests did not inactivate the fungi because the contact time, from 30 s to 8 min, turned out to be too short [29].

Ethanol at 30%, 70% and 100% was shown to have antifungal activity on historical paper. The best results were obtained for 70% ethanol, showing fungicidal properties on four out of five fungal species tested (*Aspergillus niger*, *Cladosporium cladosporioides*, *Penicillium chrysogenum* and *Penicillium corylophilum*). No harmful effects of 70% ethanol were observed on the tested paper in the short or long term [30].

Tests with 70–80% alcohol were performed for the application of ethanol on antique leather. Satisfactory results were obtained only after several hours of poultice application and soaking of the skin sample for 10 min in an aqueous alcohol solution. Good effectiveness was achieved; however, the author does not recommend this method for use on cultural heritage sites [31].

The effectiveness tests of 70% liquid ethanol on vintage paper showed that it is not sporicidal [32].

The lack of good effectiveness of ethanol in liquid form on Bacillus spores is confirmed by data provided in the literature [33].

Due to the possibility of damaging a historical object by using an aqueous solution of ethanol in this study, an attempt was made to use it in the form of mist, optimizing both the concentration, pressure and distance of the airbrush from the object; the weight of the applied agent; and the time of application.

Karbowska tested the biocidal effectiveness in ethanol vapors applied for 18 h on old paper and showed a good reduction of the tested fungi (R > 4.00 log). In the case of *Cladosporium cladosporioides*, only a 3 h exposure to ethanol vapor was sufficient. Other fungi, including *Penicillium spinulosum* and the most resistant strains of *Trichoderma viride* and *Chaetomidium subfimeti* were eliminated after 18 h [14].

In this study, the use of ethanol in the form of mist on contemporary and historical cotton from the A-BSM resulted in a reduction of more than 99% in the number of microorganisms.

The *Bacillus subtillis* bacteria, after using 90% ethanol in the form of mist and 22 h of storage, was reduced to the smallest extent among all the tested microorganisms (73%), which did not give the expected reduction, but it is a better result than after using ethanol in the form of a liquid. A worse reduction was achieved than with VHP and a laser; however, there is no better technique to apply to places with visible biodeterioration that occupy a small area of the facility and are not large enough to disinfect the entire facility. The low effectiveness of ethanol against *Bacillus subtilis* was due to the fact that most of it occurred in the form of spores, and the adverse effect of ethanol on the growth, viability and metabolism of bacteria was caused primarily by leakage of the plasma membrane. In this study, an attempt was made to reduce B. subtillis from the fabric surface using 60% and 80% ethanol solutions. A better reduction was achieved using a lower concentration because the higher water content in the solution, due to slower evaporation, extended the time of direct contact of the disinfectant with microorganisms. The growth tolerance of various microorganisms to ethanol was largely due to adaptive and evolutionary changes in cell membrane compositions. Different cellular activities differ in ethanol tolerance [34].

The reduction obtained for strains of *S. aureus*, *A. flavus* and *A. niger* after 4 s of application and 22 h of storage in foil was the highest and ranged between 99.9% and 100%. However, differences in reduction values occurred between species, which were most likely due to the different microbial growth on the control cotton fabric that was not treated with ethanol.

The main objective of the work was to demonstrate the biocidal effectiveness of ethanol in the form of mist on historical materials made of fabric and its impact on contemporary cotton. However, in museology, the adaptation of new disinfection techniques must be associated with an analysis of the consequences of such a process. Therefore, the assessment of changes was performed using SEM. This method is commonly used for the detailed analysis of cotton structure morphology [35].

The SEM images did not show any greater heterogeneity of the samples after the use of ethanol on the model and historical cotton, which is a very good result because damage and cracks would weaken the object and provide an additional place for the adhesion of microorganism cells.

Each attempt to use disinfectants should be preceded by validation of the method, which was confirmed by these studies. An example may be a situation where, despite the use of the same airbrush pressure, i.e., 0.2 MPa, and the PA HEAD VLH-5 nozzle, the application time with another device had to be extended to achieve the appropriate mass of the applied ethanol.

It is important not to apply too much ethanol to museum objects since this could cause changes in the structure and deform the object by shrinking the threads, and thus, contribute to their biodeterioration.

## 5. Conclusions

Disinfection with ethanol in the form of mist with optimized concentrations and application times and techniques showed biocidal effectiveness and can be used on historical objects made of cotton fabrics because it does not damage the surface structure. Like any implemented method for use on historic material, it requires validation for specific objects.

## Figures and Tables

**Table 1:** Evaluation of biocidal effectiveness of the short-term action of ethanol in the form of mist against *C. cladosporioides*, *A. alternata*, *S. aureus* and *A. niger* on model fabric.

Microorganism	Ethanol Concentration	Ethanol Application Time	Mass of Ethanol (g/100 cm^2^)	Number of Microorganisms (CFU/Sample)	Mean			Reduction (%)
					The Average Number of Microorganisms (CFU/Sample)	Standard Deviation	Log (CFU/Sample)	
*Cladosporium cladosporioides*	0%	0 s	0.00	1.35 × 10^5^	1.61 × 10^5^	4.68 × 10^4^	5.19	-
				2.15 × 10^5^				
				1.33 × 10^5^				
	80%	10 s	0.58	9.00 × 10^2^	1.48 × 10^3^	9.25 × 10^2^	3.12	99.08
				1.00 × 10^3^				
				2.55 × 10^3^				
	90%	9 s	0.37	<100	1.50 × 10^2^	8.66 × 10^1^	2.13	99.91
				2.50 × 10^2^				
				<100				
*Alternaria alternata*	0%	0 s	0.00	1.45 × 10^5^	1.40 × 10^5^	2.57 × 10^4^	5.14	-
				1.10 × 10^5^				
				1.60 × 10^5^				
	80%	10 s	0.77	1.77 × 10^3^	1.10 × 10^3^	8.96 × 10^2^	3.05	99.21
				1.50 × 10^3^				
				1.00 × 10^2^				
	90%	9 s	0.58	1.27 × 10^3^	2.30 × 10^3^	1.72 × 10^3^	3.37	98.36
				4.32 × 10^3^				
				1.41 × 10^3^				
*Staphylococcus aureus*	0%	0 s	0.00	6.59 × 10^4^	5.91 × 10^4^	1.93 × 10^4^	4.75	-
				7.41 × 10^4^				
				3.73 × 10^4^				
	80%	10 s	0.50	1.23 × 10^3^	1.97 × 10^3^	6.50 × 10^2^	3.28	96.67
				2.45 × 10^3^				
				2.23 × 10^3^				
	90%	9 s	0.41	3.86 × 10^3^	2.98 × 10^3^	8.85 × 10^2^	3.46	94.96
				2.09 × 10^3^				
				3.00 × 10^3^				
*Aspergillus niger*	0%	0 s	0.00	6.10 × 10^4^	5.76 × 10^4^	4.16 × 10^3^	4.76	-
				5.32 × 10^4^				
				5.86 × 10^4^				
	80%	15 s	0.61	1.81 × 10^3^	3.75 × 10^3^	1.75 × 10^3^	3.57	93.62
				4.23 × 10^3^				
				5.23 × 10^3^				
	90%	15 s	0.43	8.55 × 10^3^	3.97 × 10^3^	4.08 × 10^3^	3.60	93.27
				6.36 × 10^2^				
				2.73 × 10^3^				

**Table 2:** Evaluation of the biocidal effectiveness of the short-term effect of ethanol in the form of mist against *Bacillus subtilis* on a model fabric.

Microorganism	Ethanol Concentration	Mass of Ethanol (g/100 cm^2^)	Number of Microorganisms (CFU/Sample)	Mean			Reduction
				The Average Number of Microorganisms (CFU/Sample)	Standard Deviation	Log (CFU/Sample)	(%)
*Bacillus subtilis*	0%	0.00	1.66 × 10^6^	1.88 × 10^6^	2.37 × 10^5^	6.27	-
		0.00	1.84 × 10^6^				
		0.00	2.13 × 10^6^				
	60%	0.84	1.50 × 10^6^	1.1 × 10^6^	3.45 × 10^5^	6.04	41.49
		0.93	8.50 × 10^5^				
		0.88	9.73 × 10^5^				
	70%	0.66	1.50 × 10^6^	1.22 × 10^6^	2.54 × 10^5^	6.08	35.11
		0.64	1.01 × 10^6^				
		0.62	1.14 × 10^6^				
	80%	0.41	1.73 × 10^6^	1.6 × 10^6^	1.96 × 10^5^	6.21	14.90
		0.53	1.73 × 10^6^				
		0.50	1.39 × 10^6^				
	90%	0.39	1.60 × 10^6^	1.50 × 10^6^	2.40 × 10^5^	6.17	20.21
		0.39	1.68 × 10^6^				
		0.40	1.23 × 10^6^				

**Table 3:** Evaluation of the long-term effectiveness of 80% ethanol applied for 10 s and 90% ethanol applied for 9 s on the model fabric and samples stored for 22 h in a closed package.

Microorganism	Ethanol Concentration	Mass of Ethanol (g/100 cm^2^)	Number of Microorganisms (CFU/Sample)	Mean			Reduction	
				The Average Number of Microorganisms (CFU/Sample)	Standard Deviation	Log (CFU/Sample)	Log (CFU/Sample)	(%)
*Aspergillus niger*	0%	0.00	1.80 × 10^5^	1.47 × 10^5^	3.82 × 10^4^	5.17	-	-
		0.00	1.05 × 10^5^					
		0.00	1.55 × 10^5^					
	80%	0.77	<100	<100	0.00	2.00	3.17	99.93
		0.87	<100					
		0.82	<100					
	90%	0.52	< 100	<100	0.00	2.00	3.17	99.93
		0.41	<100					
		0.46	<100					
*Aspergillus flavus*	0%	0.00	3.59 × 10^5^	2.85 × 10^5^	6.54 × 10^4^	5.45	-	-
		0.00	2.36 × 10^5^					
		0.00	2.59 × 10^5^					
	80%	0.68	<100	<100	0.00	2.00	3.45	99.96
		0.69	<100					
		0.68	<100					
	90%	0.40	<100	<100	0.00	2.00	3.45	99.96
		0.47	<100					
		0.42	<100					
*Staphylococcus aureus*	0%	0.00	5.18 × 10^6^	6.41 × 10^6^	1.68 × 10^6^	6.81	-	-
		0.00	5.73 × 10^6^					
		0.00	8.32 × 10^6^					
	80%	0.59	<100	<100	0.00	2.00	4.81	100.00
		0.54	<100					
		0.52	<100					
	90%	0.49	<100	<100	0.00	2.00	4.81	100.00
		0.52	<100					
		0.51	<100					
*Bacillus subtilis*	0%	0.00	2.80 × 10^5^	2.52 × 10^5^	1.40 × 10^5^	5.40	-	-
		0.00	2.48 × 10^5^					
		0.00	2.29 × 10^4^					
	80%	0.73	8.91 × 10^4^	9.01 × 10^4^	1.23 × 10^4^	4.96	0.44	73.81
		0.80	1.04 × 10^5^					
		0.90	7.95 × 10^4^					
	90%	0.61	1.03 × 10^5^	8.86 × 10^4^	1.29 × 10^4^	4.94	0.46	74.24
		0.58	8.45 × 10^4^					
		0.47	7.82 × 10^4^					

**Table 4:** Evaluation of the effectiveness of long-term action of ethanol applied for 4 s on a model fabric.

Microorganism	Ethanol Concentration	Mass of Ethanol (g/100 cm^2^)	Number of Microorganisms (CFU/Sample)	Mean			Reduction	
				The Average Number of Microorganisms (CFU/Sample)	Standard Deviation	Log (CFU/Sample)	Log (CFU/Sample)	(%)
*Aspergillus niger*	0%	0.00	1.60 × 10^5^	1.28 × 10^5^	4.65 × 10^5^	5.11	-	-
		0.00	1.30 × 10^5^					
		0.00	9.50 × 10^5^					
	80%	0.38	<100	<100	0.00	2.00	3.11	99.92
		0.38	<100					
		0.36	<100					
	90%	0.29	<100	<100	0.00	2.00	3.11	99.92
		0.28	<100					
		0.27	<100					
*Aspergillus flavus*	0%	0.00	2.27 × 10^5^	2.96 × 10^5^	9.00 × 10^3^	5.42	-	-
		0.00	2.36 × 10^5^					
		0.00	2.45 × 10^5^					
	80%	0.35	<100	<100	0.00	2.00	3.42	99.96
		0.33	<100					
		0.42	<100					
	90%	0.31	<100	<100	0.00	2.00	3.42	99.96
		0.32	<100					
		0.26	<100					
*Staphylococcus aureus*	0%	0.00	3.86 × 10^6^	3.60 × 10^6^	1.50 × 10^6^	6.52	-	-
		0.00	1.98 × 10^6^					
		0.00	4.95 × 10^6^					
	80%	0.32	<100	<100	0.00	2.00	4.52	100.00
		0.33	<100					
		0.29	<100					
	90%	0.24	<100	<100	0.00	2.00	4.52	100.00
		0.24	<100					
		0.25	<100					

**Table 5:** Evaluation of the effectiveness of the long-term effect of ethanol applied for 4 s on antique fabrics.

Microorganism	Ethanol Concentration	Mass of Ethanol (g/100 cm^2^)	Number of Microorganisms (CFU/Sample)	Log (CFU/Sample)	Reduction	
					Log (CFU/Sample)	(%)
*Staphylococcus aureus* ATCC	90%	0.30	<100	2.00	4.41	100.00
*Apergillus flavus* ATCC	90%	0.27	<100	2.00	3.43	99.96
*Apergillus niger* ATCC	90%	0.27	<100	2.00	3.25	99.95
*Apergillus niger* (isolated from a historical object)	90%	0.29	<100	2.00	3.16	99.93
*Penicillium chrysogenum* (isolated from a historical object)	90%	0.27	<100	2.00	2.88	99.87
*Alternaria alternata* ATCC	0%	0.00	1.64 × 10^5^	5.21	3.21	99.94
	90%	0.45	<100	2.00		

## Data Availability

No new data has been created.
